# Physical Selection and Verification of a Trusted Source Signal Using an Angle-Selective Metasurface

**DOI:** 10.3390/s26196055

**Published:** 2026-09-24

**Authors:** Kaisarali Kadyrzhan, Bernur Karimzhan, Dina Shaltykova, Yerkebulan Nurgizat, Ibragim Suleimenov

**Affiliations:** 1 National Engineering Academy of the Republic of Kazakhstan, Almaty 050010, Kazakhstan; kaisarali1997ss@gmail.com (K.K.); shaltykova.d@mail.ru (D.S.); esenych@yandex.kz (I.S.); 2 Department of Electronic Engineering, Almaty University of Power Engineering and Telecommunications Named After Gumarbek Daukeyev, Almaty 050062, Kazakhstan; y.nurgizat@aues.kz; 3 Institute of Digital Sciences and Artificial Intelligence, L.N. Gumilyov Eurasian National University, Astana 010008, Kazakhstan

**Keywords:** physical level of information security, UAV, trusted radio signal source, metasurface, angular selectivity, frequency-selective surface, signal selection, physical authentication, HFSS

## Abstract

The development of physical-level information security methods allows us to supplement traditional cryptographic tools with instruments directly related to signal propagation conditions and the spatial location of a signal’s source. Previous work by the authors demonstrated that when transmitting commands to a group of unmanned aerial vehicles (UAVs), the coordinates of a trusted radio transmitter or the direction to it can be determined based on joint measurements by several UAVs during secure data exchange within the group, for example, over fiber-optic lines. For practical purposes, protecting a command channel requires high localization accuracy: if the operator’s location is known a priori and the system operates within direct radio visibility, it is sufficient to establish that the received signal belongs to a trusted angular sector. However, determining the direction alone does not solve the problem of selecting the desired signal in the presence of intentional jamming or spoofed signals. In this paper, we propose the use of an angle-selective metasurface for the second level of protection. A two-channel scheme with a reference receiving channel and a channel with a flat metasurface is proposed. A periodic unit cell for the 6 GHz band was developed, and full-wave simulations were performed in Ansys HFSS. A pronounced *S*_21_(*f*, *θ*) dependence was obtained in the 5.8–6.2 GHz band, with incidence angles varying from 0° to 15°. The maximum local contrast is approximately 5.67 dB, and the band-averaged absolute change relative to normal incidence is approximately 2.1–4.7 dB. It is shown that due to the non-monotonicity of *S*_21_(*f*, *θ*), the metasurface should be used not as a standalone absolute angle meter but as a physical selector and signal verifier after pre-determining the trusted direction.

## 1. Introduction

Protecting information during command transmission to unmanned aerial vehicles (UAVs) has traditionally been associated primarily with cryptography, error-correcting coding, and secure network protocols. However, the development of next-generation wireless systems increasingly demonstrates the feasibility of supplementing these tools with physical-layer security (PLS). This promising additional layer of protection is becoming particularly important, for example, for low-power and computationally limited IoT devices and future 6G systems [1,2,3]. Optical and fiber-optic technologies occupy a special place in this trend. Modern literature considers the physical layer of optical networks as an independent object of protection against interception, signal injection, jamming, and other attacks [4,5]. Thus, the general trend is toward a transition from a purely software-cryptographic interpretation of security to a multi-layer architecture in which the physical properties of channels and signals play a significant role.

This approach was applied by the authors of this paper to the problem of securely transmitting commands to a group of UAVs. In [6,7], an architecture is proposed in which data received by various devices in a group can be transmitted between them via fiber-optic lines, and the position of a remote radio transmitter is determined based on joint measurements. The method is designed for operation within line of sight, with the spatial position of the transmitter becoming not an auxiliary characteristic but part of the trusted team identification procedure.

For the practical application of this principle, it is important that high coordinate determination accuracy is not always required. If the location of a trusted operator or transmitting station is a priori limited to a known area, then for authentication purposes, it is sufficient to establish whether the source of the received signal belongs to an acceptable spatial sector. This is especially true for line-of-sight scenarios, for example, in complex jamming environments near the line of contact. The trusted control point is located in a predetermined spatial region, while the source of external interference is typically located at a considerable distance. Consequently, the task of high-precision radio direction finding can be replaced by the simpler task of classifying “trusted sector/other directions” for which classification in terms of a spatial rose of directions can be applied [8].

However, determining the trusted direction is only the first stage of physical protection. The remaining task is selecting the useful signal in the presence of extraneous radio emissions. Even if the direction to the trusted source is known, it is necessary to transform the spatial difference between it and extraneous sources into a stable, measurable feature of the receiving path.

Angle-selective metasurfaces offer a promising solution to this problem. Modern research shows that periodic electromagnetic structures can generate a pronounced dependence of the transmission coefficient on the angle of wave incidence, thereby implementing spatial filtering [9,10,11,12,13]. The development of reconfigurable smart surfaces has further led to the use of a controlled electromagnetic environment directly in physical authentication tasks [14]. In contrast to these approaches, in this paper, the metasurface is considered as a passive hardware selector, activated after preliminary localization of the trusted source.

The proposed architecture therefore has a two-stage nature. In the first stage, the trusted source position, bearing, or angular sector is determined using the previously developed source-localization and direction-sector classification methods [7,8] or another radio engineering technique. This requires reliable identification of a trusted angular sector rather than extreme accuracy. In the second stage, the receiving unit is oriented relative to the determined direction, and an angle-selective metasurface is installed in front of one of the receiving antennas. Deviation in the direction of arrival alters its frequency-dependent transfer characteristic, resulting in the spatial difference between the sources being transformed into a spectral-amplitude characteristic. The purpose of this work is to numerically substantiate the feasibility of this second stage—physical selection and verification of a signal arriving from a predetermined trusted direction.

The proposed metasurface is therefore a local verification element at the protected receiving node and is not intended to be inserted at every relay or hop of a multi-hop network. The present numerical study considers a line-of-sight (LoS) verification interval in which the trusted direction has already been established and the receiver orientation is treated as quasi-static; rapid platform attitude changes and dynamic alignment compensation are outside the scope of the present HFSS model.

## 2. Scientific Novelty and Contribution of the Work

The present work extends the previously developed approach for trusted-source localization and direction-sector classification [7,8] by introducing a second, independent physical verification stage based on an angle-selective metasurface. Previous studies addressed the determination of source coordinates, bearing, or trusted spatial sector; the present work uses that spatial information to verify whether the received signal is consistent with the trusted direction. In the present work, the previously determined trusted direction is used as prior information for a second, independent verification stage: the metasurface-loaded channel converts a deviation from this direction into an angle-dependent spectral transformation that can be compared with the reference channel.

The proposed system contains two receiving channels. The first directly records the received radio signal and serves as a reference. In the second, a flat metasurface with a frequency-angular transfer characteristic is installed in front of a similar receiving antenna. The first physical feature is generated by the preliminary localization system and is associated with the spatiotemporal or phase parameters of the received signal; the second arises from the electrodynamic interaction of the incident wave with a periodic structure. Therefore, the additional channel does not repeat the localization algorithm but performs independent hardware verification of its results. Accordingly, the specific contribution of this work is the formulation of a dual-channel receiver in which an angle-dependent spectral feature provides a local consistency check for a predetermined trusted direction. This contribution includes a dual-channel architecture, the development and parametric optimization of a periodic cell in HFSS, the calculation of transfer characteristics in the 5.8–6.2 GHz band, and the establishment of the structure’s usefulness as a selector and verifier of a predetermined direction rather than as an unambiguous measure of absolute angle.

## 3. The Concept of Two-Channel Physical Selection and Verification

It is assumed that the trusted direction or angular sector has already been determined using the previously developed source-localization and direction-sector classification methods [7,8] or another radio engineering technique. The result of the first stage may not be the exact coordinate value but a trusted angular sector. The receiving unit is oriented so that the metasurface normal coincides with the central direction of this sector. The first channel contains a receiving antenna without an angle-selective element and serves as a reference. In the second channel, a metasurface is installed in front of a similar antenna. The spectra of the output signals can be written as

*V*_1_(*f*, *θ*) = *S*(*f*)*H*_1_(*f*, *θ*), (1)

*V*_2_(*f*, *θ*) = *S*(*f*)*H*_2_(*f*, *θ*)*H*_MS_(*f*, *θ*), (2)

where *S*(*f*) is spectrum of the received signal, *H*_1_ and *H*_2_ are characteristics of antennas and receiving paths, and *H_MS_* is the transfer characteristic of a metasurface. Thus, the spatial deviation of the external source from the trusted direction is converted into a change in the spectrum of the second channel.

When calibrating for the trusted direction, further taken as *θ* = 0°, it is determined that

(3) $$D_{0}\left(f\right)=20\log_{10}\left|\frac{V_{2}\left(f,0^{{}^{\circ}}\right)}{V_{1}\left(f,0^{{}^{\circ}}\right)}\right|.$$

The working differential feature has the form of

(4) $$D\left(f,\theta \right)=20\log_{10}\left|\frac{V_{2}\left(f,\theta \right)}{V_{1}\left(f,\theta \right)}\right|-D_{0}\left(f\right).$$

The transformation introduced by the metasurface is therefore informative. This is crucial for selection under external interference: a signal from a different angular sector should produce a different frequency-angular signature, even with comparable absolute power. The decision threshold is not fixed a priori; it should be determined after experimentally assessing noise, drift, multipath, and the spread of the receiving paths.

For clarity, the verification procedure can be summarized as follows: (1) determine the trusted direction or angular sector using the preceding localization stage, (2) acquire the spectra of the reference channel and the metasurface-loaded channel, (3) normalize the metasurface-induced response relative to the trusted-direction calibration state, (4) form the broadband differential spectral signature, and (5) compare the observed signature with the calibrated trusted-direction signature and use the result as an additional physical consistency criterion before conventional protocol or cryptographic verification.

## 4. Angle-Selective Metasurface as a Physical Selector

To implement the second physical channel, a periodic metasurface was developed to generate a pronounced frequency-angular dependence of the transmission coefficient at approximately 6 GHz. In the system under consideration, the angular dependence is not suppressed but is deliberately used as an informative indicator of the received signal’s belonging to a trusted sector. The printed implementation on a thin dielectric layer belongs to the general class of frequency-selective and high-impedance periodic surfaces, the analysis methods for which are well-developed [15].

The metasurface is positioned in front of the antenna of the second receiving channel so that its normal coincides with the expected direction of the trusted source. Changing the direction of arrival alters the transmission coefficient (*S*_21_(*f*, *θ*)), i.e., the spatial deviation is converted into a change in the parameters recorded by the second channel. We emphasize that this does not involve complete suppression of any signal outside a given angle but the formation of a stable additional indicator suitable for comparison with the reference channel. For the fundamental Floquet mode, the transmission coefficient is determined by the standard ratio of *S*_21_(*f*, *θ*) = *b*_2_/*a*_1_, where a_1_ is the amplitude of the incident mode at the input Floquet port and *b*_2_ is the amplitude of the transmitted mode at the output Floquet port for the same frequency (*f*) and incidence angle (*θ*). This definition follows the standard power-wave scattering-matrix convention [16].

The unit cell of the metasurface contains mutually orthogonal metallic resonant elements. Geometric symmetry was chosen as a prerequisite for reducing dependence on the orientation of linear polarization. However, in this study, the primary goal of the modeling was angular selectivity; polarization independence must be confirmed by a separate numerical or experimental study.

Full-wave simulation of a periodic unit cell was performed in Ansys HFSS, part of Ansys Electronics Desktop 2025 R2.4 (Ansys, Inc., Canonsburg, PA, USA). Coupled periodic lattice-pair conditions were imposed on opposite side faces of the computational domain, and the upper and lower boundaries were excited by Floquet ports. The ports accounted for two fundamental orthogonal modes. The azimuth angle in this series of simulations was set to *φ* = 0°, and the angle *θ* varied relative to the surface normal.

The parameters listed in Table 1 were selected as a result of a parametric analysis of the geometric dimensions. The unit cell period is determined by the *a* parameter. The *L*_1_ parameter determines the length of the outer horizontal arm of the metallic resonant element along the *x*-axis, while *L*_2_ determines the length of the inner horizontal arm. The *w* parameter corresponds to the width of the metal strip, *g* corresponds to the central gap between the symmetrical halves of the structure, *h* corresponds to the thickness of the dielectric substrate, and *t*Cu corresponds to the thickness of the copper layer. When moving to physical prototyping, the choice of a specific microwave dielectric should be made taking into account the compliance of its electrophysical parameters with the calculation model; typical data for high-frequency laminates of the RO4000 family are given, for example, in the technical documentation of Rogers Corporation [17]. The selected unit-cell geometry, layer stack, and periodic repetition are summarized in Scheme 1.

The initial analysis was performed over an extended frequency range to localize resonant features. A detailed study was conducted in the operating band of 5.8–6.2 GHz. The incidence angle was varied in the range of 0–15°. The frequency response was calculated using an interpolation sweep. The fundamental Floquet mode transmission coefficient (*S*_21_(*f*, *θ*)) was used as the primary output variable.

The adaptive solution was performed at a frequency of 6 GHz. No more than 15 passes were used for mesh adaptation, with a convergence criterion of maximum *ΔS* = 0.02. For the base variation of *θ* = 0°, *φ* = 0°, convergence was achieved in nine adaptive passes; in the final pass, maximum *ΔS* = 0.013737, i.e., below the specified threshold. The final adaptive mesh contained 4615 tetrahedral elements, including 496 Metal_Top elements, 2674 RadiatingSurface elements, and 1445 Substrate elements. Frequency dependencies were calculated using the interpolating sweep method; the final series used the 5.8–6.6 GHz range with 401 points, and the working region analysis was performed in the 5.8–6.2 GHz band. The *θ* angle was specified using the scan angles of periodic lattice pairs, with *φ* = 0°. The final angular series included *θ* = 0–15° with a step of 1°. This setup and the given convergence parameters allow for reproduction of the numerical calculation using the same geometry, materials, Floquet modes, and periodic boundary conditions.

Parametric optimization was performed sequentially, taking into account the physical role of geometric parameters in shaping the resonant response (*S*_21_(*f*)). *L*_1_ was chosen as the first variable parameter, since it directly determines the length of the outer horizontal conductive arm of the resonant element and, thus, significantly affects the effective electrical length of the current path and the location of resonant features. In the first stage, the sensitivity of the frequency response to changes in *L*_1_ was investigated. As can be seen from Figure 1, changing *L*_1_ restructures the frequency response and shifts the region of angle-sensitive behavior. Of the options considered, *L*_1_ = 7.00 mm ensures the most informative changes in *S*_21_ are located near the required range of approximately 6 GHz and was therefore retained for the next stage of optimization. For readability, Figure 1, Figure 2 and Figure 3 show the corresponding parameter sweeps for *θ* = 0°, 4°, 8°, 12°, and 15°, reconstructed from the original exported HFSS datasets.

Figure 1 confirms that *L*_1_ is a key tuning parameter of the resonant region: varying *L*_1_ shifts the positions of the maxima and minima of *S*_21_(*f*) and moves the angle-sensitive spectral region. After selecting *L*_1_ = 7.00 mm, the second linear parameter *L*_2_ was used to tune the spectral position of the angle-sensitive response more precisely.

After fixing *L*_1_ = 7.00 mm, the length of the inner horizontal arm (*L*_2_) was varied. As Figure 2 shows, changing *L*_2_ shifts the characteristic features of *S*_21_(*f*), which is associated with a change in the effective electrical length of the resonator’s inner section and its electromagnetic coupling with the rest of the structure. A value of *L*_2_ = 3.70 mm was adopted for subsequent adjustment of the central gap (*g*).

During the final stage of geometric optimization, the gap (*g*) was varied. Figure 3 shows that this parameter significantly influences the position and depth of the resonant minima. The value *g* = 1.60 mm was retained in the final geometry, after which a detailed angular sweep of 0–15° was performed.

## 5. Results of Electrodynamic Modeling and Analysis of Angular Selectivity

After fixing the geometry, the main question was whether the difference in the metasurface response was maintained with angle changes across the entire 5.8–6.2 GHz operating band. Therefore, the final calculation was performed not for individual test angles but for *θ* = 0–15° in 1° increments. A normalized characteristic was used for comparison with normal incidence.

*ΔS*_21_(*f*, *θ*) = *S*_21_(*f*, *θ*) − *S*_21_(*f*, 0^°^). (5)

Here, *ΔS*_21_(*f*, *θ*) is the normalized change in the transmission coefficient in decibels relative to the calibration state at normal incidence; *S*_21_(*f*, *θ*) is the calculated transmission coefficient at frequency *f* and incidence angle *θ*; *S*_21_(*f*, *0°*) is the transmission coefficient at the same frequency at normal incidence. Thus, *ΔS*_21_(*f*, *θ*) directly characterizes the additional change in the transmission characteristic caused by the angular deviation from the trusted direction. Representative spectra from the full angular sweep are shown in Figure 4; all simulated angles remain included in the two-dimensional map and band-averaged analysis. The curves do not coincide: both the position of the narrow minima and the overall level in the band under consideration change. Consequently, the angle of arrival is encoded not by a single transmission value but by the shape of the frequency response. In the studied frequency-angular region, the maximum local contrast reaches approximately 5.67 dB. The frequency tuning is most clearly demonstrated in Figure 4. For different S_21_ values, the resonant features shift in frequency and change in depth. This is an important result: selectivity is not tied to a single pre-selected frequency but manifests itself as a change in the spectral signature. To verify the operating bandwidth, the same data are presented in a different section—as a dependence on the angle at fixed frequencies of 5.8, 5.9, 6.0, 6.1, and 6.2 GHz (Figure 5).

Figure 5 confirms that changing the angle results in a measurable change in the transmission coefficient at all five test frequencies in the operating band. The magnitude and sign of the change depend on frequency, while the dependence on *θ* is not strictly monotonic. This is why broadband processing is preferable for the proposed verifier: the combined analysis of multiple frequencies allows for the use of a characteristic spectral–angular signature instead of a single scalar level. To compactly represent the entire HFSS dataset, a two-dimensional map (*S*_21_(*f*, *θ*)) was constructed, as shown in Figure 6.

The map in Figure 6 allows one to track the characteristics restructuring simultaneously along two coordinates—frequency and angle. To reduce this information to a single quantitative indicator suitable for comparing angles, an integral characteristic was introduced:

(6) $$A\left(\theta \right)=\frac{1}{B}\int_{f_{1}}^{f_{2}}\left|\Delta S_{21}\left(f,\theta \right)\right|\;\mathrm{df},$$

where *A*(*θ*) is the average absolute change in the transmission coefficient relative to normal incidence over the operating band; *f*_1_ = 5.8 GHz and *f*_2_ = 6.2 GHz are the lower and upper boundaries of the operating band, respectively; *B* = *f*_2_ − *f*_1_ = 0.4 GHz is the width of the operating band; and |*ΔS*_21_(*f*, *θ*)| is the modulus of the normalized characteristic defined by expression (5). Thus, *A*(*θ*) is a single broadband quantitative indicator of angular sensitivity. The obtained dependence is shown in Figure 7. For the studied non-zero angles, the average absolute change relative to *θ* = 0° is approximately 2.1–4.7 dB, and the maximum value of about 4.67 dB is observed at *θ* = 6°. Thus, even after averaging over the entire band, the angular effect does not disappear, which confirms the suitability of the structure for forming an additional directional feature.

A combined analysis of Figure 4, Figure 5, Figure 6 and Figure 7 leads to the key conclusion of the simulation. The metasurface does exhibit angular selectivity in the required 5.8–6.2 GHz band, but this selectivity is spectral rather than simply monotonic. Therefore, using a single *S*_21_ value to unambiguously determine absolute *θ* is impractical. For the purpose of this study, this is not a drawback: the metasurface should not measure the angle itself but verify the direction already found by the main method using an additional frequency-angular signature.

To further interpret the angular dependence, the distribution of the electric field modulus |E| in the substrate at a frequency of 6.0 GHz was calculated for *θ* = 0° and *θ* = 6°. Both maps in Figure 8 are presented on a common linear scale of 0–6500 V/m, so the intensities can be compared directly. At normal incidence, the field is localized primarily near the internal bends and gaps of the resonant elements, and the maximum value reaches 6433.59 V/m. At *θ* = 6°, the maximum decreases to 2768.80 V/m, i.e., to 43.0% of the value at *θ* = 0°; the relative reduction is approximately 57.0%, or 2.32 times.

The change in the local field is consistent with the angular tuning of the transmission coefficient. At 6.0 GHz, the transition from *θ* = 0° to *θ* = 6° is accompanied by a change in *S*_21_ of approximately 5.38 dB, simultaneously with a significant decrease in the maximum local field strength. The coincidence of these two features supports the interpretation of angular selectivity as a consequence of angle-dependent excitation of the resonant electromagnetic response of the unit cell. However, |*E*| maps are not used here to establish a specific intrinsic modal mechanism; such a conclusion would require a separate modal or current analysis.

## 6. Circuit Implementation, Selection and Signal Processing

The radio frequency component can be implemented with two parallel receiving paths or with a common measurement path and microwave switch. In the latter case, using a common amplifier and detector reduces the impact of gain differences and temperature drift. For broadband selection, it is preferable to compare *D*(*f*, *θ*) across several frequency samples and match the observed spectral signature with the calibration signature of the trusted sector.

In a simplified implementation, the broadband signature can be formed from the integrated powers of two receiving channels, calculated using expressions (7) and (8).

(7) $$P_{1}=\int_{f_{1}}^{f_{2}}{\left|V_{1}\left(f\right)\right|}^{2}\;\mathrm{df},$$

(8) $$P_{2}=\int_{f_{1}}^{f_{2}}{\left|V_{2}\left(f\right)\right|}^{2}\;\mathrm{df}.$$

After calibration for the trusted direction of *θ* = 0°, the normalized energy feature defined by expression (9) is used.

(9) $$D_{P}=10\log_{10}\left(\frac{P_{2}}{P_{1}}\right)-D_{P0}.$$

In expressions (7) and (8), *P*_1_ and *P*_2_ are the integrated signal powers, respectively, in the reference channel and in the channel with the metasurface in the [*f*_1_, *f*_2_] band, *V*_1_(*f*) and *V*_2_(*f*) are the complex spectral amplitudes of the signals at the output of the corresponding receiving paths. In expression (9), *D_p_* is the normalized wideband differential feature in decibels, and *D_p_*_0_ is the calibration value, i.e., 10*log*_10_(*P*_2_/*P*_1_), measured for the trusted direction of *θ* = 0°. Full spectral processing (*D*(*f*, *θ*)) contains more information, while the *D_p_* indicator allows for simplification of the hardware implementation. The metasurface is not used as a standalone absolute angle meter: its purpose is to form an additional physical feature that distinguishes a signal consistent with the trusted direction from spatially separated extraneous radiation. The final solution may combine this feature with the initial localization result and, if necessary, with cryptographic message verification.

## 7. Proposed Methodology for Future Experimental Verification of Physical Selection

The next step is experimental verification of the final panel. The basic setup includes a transmitting antenna, a radio-frequency signal source, the metasurface under study, a receiving antenna, and a transmission coefficient measurement device. The metasurface and receiving antenna are mounted on a rotating platform. In the first step, the calculated angular response is reproduced, and the experimental calibration signature of the trusted direction is determined.

Measurements are best performed in the 5.8–6.2 GHz band at *θ* = 0–15° in 1° increments. For each position, the experimental transmission coefficient (*S*_21_*exp*(*f*, *θ*)) is measured, after which the normalized experimental response is calculated relative to the calibration state (*θ* = 0°) using expression (10).

(10) $$\Delta S_{21}^{\exp}\left(f,\theta \right)=S_{21}^{\exp}\left(f,\theta \right)-S_{21}^{\exp}\left(f,0^{{}^{\circ}}\right).$$

In expression (10), *S*_21_*exp*(*f*, *θ*) is the experimentally measured transmission coefficient of the final sample at frequency *f* and angle *θ*, *S*_21_*exp*(*f*, *0°*) is the experimental calibration characteristic at normal incidence, and *ΔS*_21_*exp*(*f*, *θ*) is their difference in decibels. A second experiment is then performed with two adjacent receiving channels. To test the selection function specifically, it is advisable to introduce a second transmitter into the setup: one source is located in the trusted sector, while the other is sequentially shifted in angle and changes in power. This setup will allow us to determine the probability of correct recognition of the trusted signal, the minimum detectable angular separation, and the stability of the solution at comparable levels of the useful and unwanted signals.

Separate measurement cycles are necessary to test the influence of polarization, for example, at *ψ* = 0°, 45°, and 90°, as well as multipath propagation. This is required because the current sweep confirms angular selectivity but does not independently prove polarization independence or noise immunity in a real environment. The two-source experiment should therefore be considered a key test of the transition from the electrodynamic principle to a system for physically selecting a trusted radio signal.

## 8. Discussion

To correctly evaluate the results, it is important to compare the developed structure with modern angle-selective surfaces and frequency-selective metasurfaces. Table 2 shows that the most powerful known solutions outperform the present model in terms of suppression depth, width of the confirmed angular range, and experimental verification. The contribution of this work lies elsewhere: the angle-selective transfer function is used as a second, physically independent channel for signal verification and selection after predetermining the trusted direction, rather than as a standalone spatial filter.

The comparison shows that the claimed novelty should not be formulated as record-breaking angular selectivity or polarization independence. A more correct formulation consists of using the measured function (*S*_21_(*f*, *θ*)) in a dual-channel architecture as an additional physical-layer feature. The reference channel estimates the overall level of the received signal, while the metasurface channel introduces an angle-dependent spectral transformation. As a result, the solution is based on a differential feature that can be used to select a signal consistent with the trusted spatial sector.

The security methods suggested by the reviewer address complementary decision variables. The NJS method in [18] detects and approximately localizes jammers from wireless-medium observations in a multi-hop IoT network, whereas SecureLink [19] authenticates UAV devices by fusing cross-layer RF and onboard-sensor fingerprints. The present method does not replace either approach: it tests whether the received signal is physically consistent with a predefined trusted arrival direction by means of a local angle-dependent spectral transformation. These mechanisms can therefore be combined in a multi-layer protection architecture.

Simulation shows that the optimized structure produces a pronounced frequency-angle dependence in a 400 MHz bandwidth. The maximum local contrast of approximately 5.67 dB and the average absolute change of approximately 2.1–4.7 dB provide a measurable margin for dual-channel verification. Additional field analysis at 6.0 GHz shows that when moving from *θ* = 0° to *θ* = 6°, the maximum |*E*| in the substrate, the intensity decreases from 6433.59 to 2768.80 V/m (approximately 57%). At the same time, the non-monotonic nature of the dependence limits the use of a single level as an estimate of the absolute angle and makes spectral signature analysis preferable.

These numerical values support the limited electromagnetic claim that the modeled periodic surface provides a measurable direction-dependent feature. They do not, by themselves, establish the authentication probability, jammer rejection probability, bit-error rate, or throughput of a complete security receiver.

We emphasize, however, that the numerical model corresponds to an infinite periodic grating. A physical panel of finite size will additionally experience edge diffraction, the influence of the distance to the receiving antenna, manufacturing tolerances, and the actual substrate parameters. Therefore, the presented numerical results should be considered a proof of principle and a basis for experimental design and not a substitute for measurements on the final design. Verification in the simultaneous presence of trusted and third-party sources is especially important.

Polarization independence has also not yet been confirmed by a separate series of calculations. Geometric symmetry is a prerequisite for reducing polarization sensitivity, but the final conclusion should be based on TE/TM and/or polarization rotation in the modeling and experiment.

The numerical convergence of the base variation was further verified using an adaptive solution: the calculation converged in nine runs with maximum *ΔS* = 0.013737 < 0.02, and the final mesh contained 4615 tetrahedral elements. The remaining significant tests are a polarization sweep and an experimental evaluation of the two-source mode. Until these tests are completed, neither the polarization independence of the structure nor the quantitative noise immunity of the system are claimed in this paper. The proposed metasurface should be considered an additional physical barrier in a multi-level security system, not a universal replacement for cryptographic methods.

## 9. Conclusions

A dual-channel architecture for physical selection and verification of radio signals is proposed, expanding on previously proposed methods for determining coordinates, bearing, or a trusted direction sector [7,8]. The key system concept is to divide the task into two stages: a relatively coarse determination of a trusted spatial sector and subsequent independent hardware verification of the received signal’s belonging to this sector using an angle-selective metasurface. This approach reduces the requirements for primary localization accuracy and focuses the metasurface on physical signal selection in the presence of extraneous radio emissions rather than on independent direction finding.

To implement the second stage, a periodic cell in the 6 GHz band was developed and parametrically optimized. Full-wave simulation in Ansys HFSS with lattice-pair and Floquet ports revealed a pronounced *S*_21_(*f*, *θ*) dependence in the 5.8–6.2 GHz band at *θ* = 0–15°. The optimized parameters are *a* = 20.32 mm, *L*_1_ = 7.00 mm, *L*_2_ = 3.70 mm, *w* = 0.97 mm, *g* = 1.60 mm, and *h* = 0.88 mm. The maximum local contrast in the studied region is approximately 5.67 dB, and the band-averaged absolute change relative to normal incidence for non-zero angles is approximately 2.1–4.7 dB. At 6.0 GHz, as *θ* changes from 0° to 6°, the maximum |*E*| in the substrate decreases from 6433.59 to 2768.80 V/m, which accompanies the angular change in *S*_21_.

The resulting characteristic is not strictly monotonic; therefore, the metasurface is proposed to be used as a source of a broadband spectral–angular signature, rather than as an unambiguous measure of absolute angle. The next step is to fabricate the final surface and experimentally verify not only *S*_21_(*f*, *θ*) and polarization sensitivity but also the dual-source mode, which directly models third-party interference. Unlike known angle-selective surfaces, which focus primarily on spatial filtering, interference suppression, and beamforming, the proposed architecture couples the angle-selective surface with the pre-localization of a trusted source and uses it as an independent hardware selector. The sequence of “trusted sector–physical signature–selection/command verification” is the main systemic contribution of this work. Accordingly, the present results should be interpreted as a numerical proof of the electromagnetic feature rather than as experimental validation of the complete security receiver.

The proposed mechanism should be used in conjunction with other security layers. If a third-party transmitter is located in the same angular sector as the trusted one, a spatial feature alone is insufficient; in this case, a combination of angle selection and additional physical, temporal, hardware, or cryptographic features is required.

## Figures and Tables

**Scheme 1:**
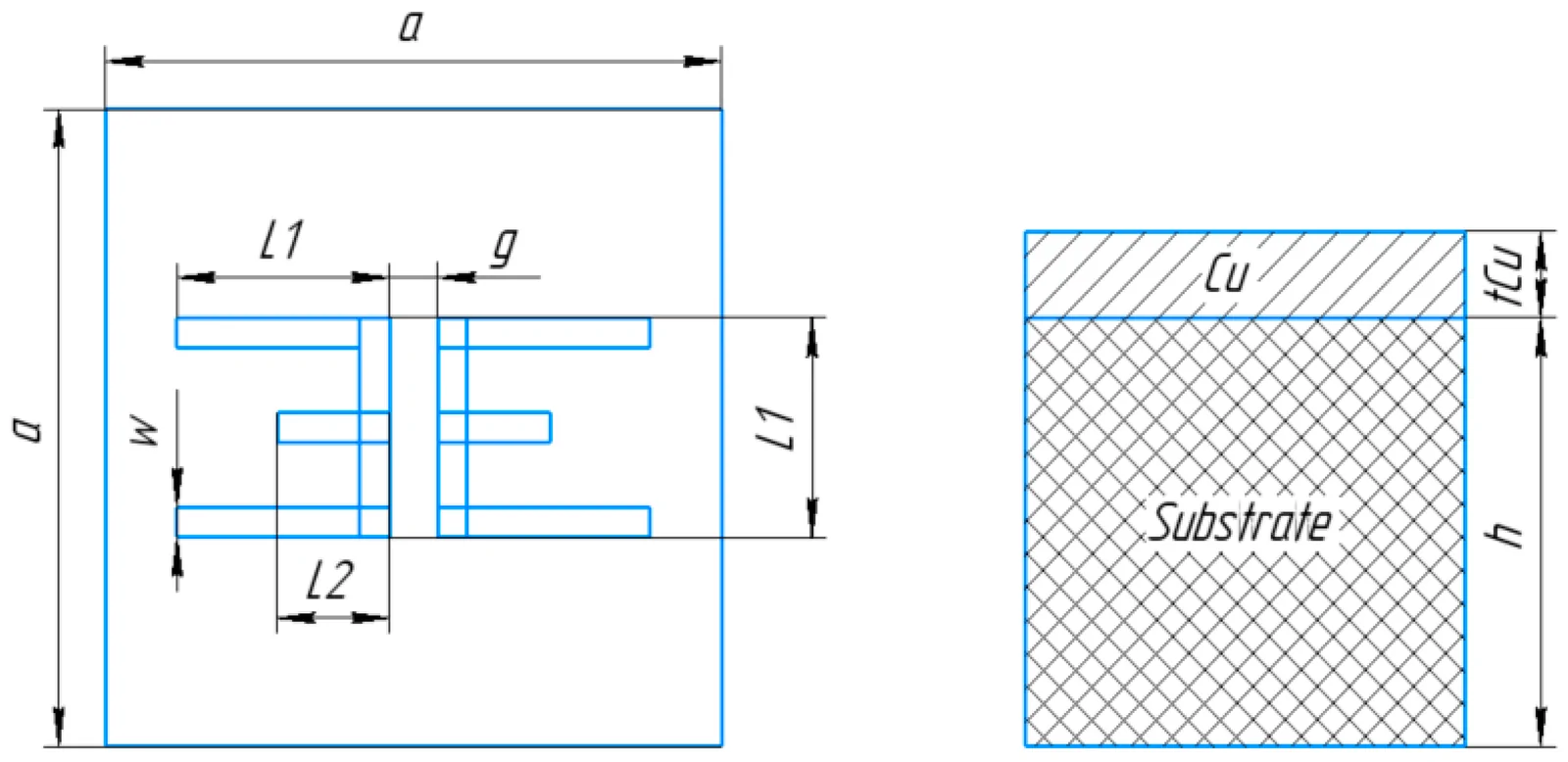
Geometry of the selected unit cell.

**Figure 1:**
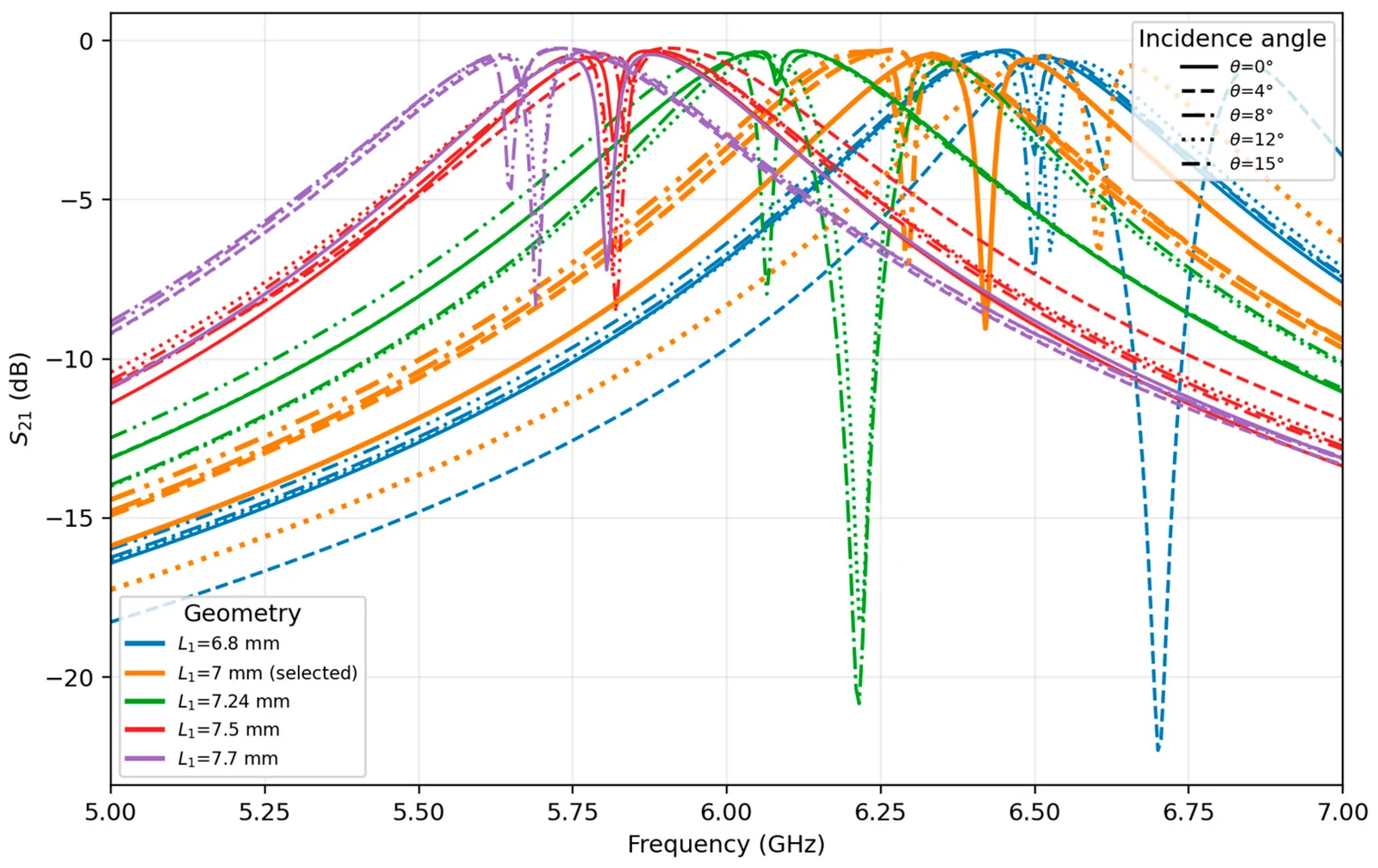
Influence of *L*_1_ on the simulated transmission response (*S*_21_(*f*)) for *θ* = 0°, 4°, 8°, 12°, and 15°. The selected value is *L*_1_ = 7.00 mm. All curves were reconstructed from the original exported HFSS data.

**Figure 2:**
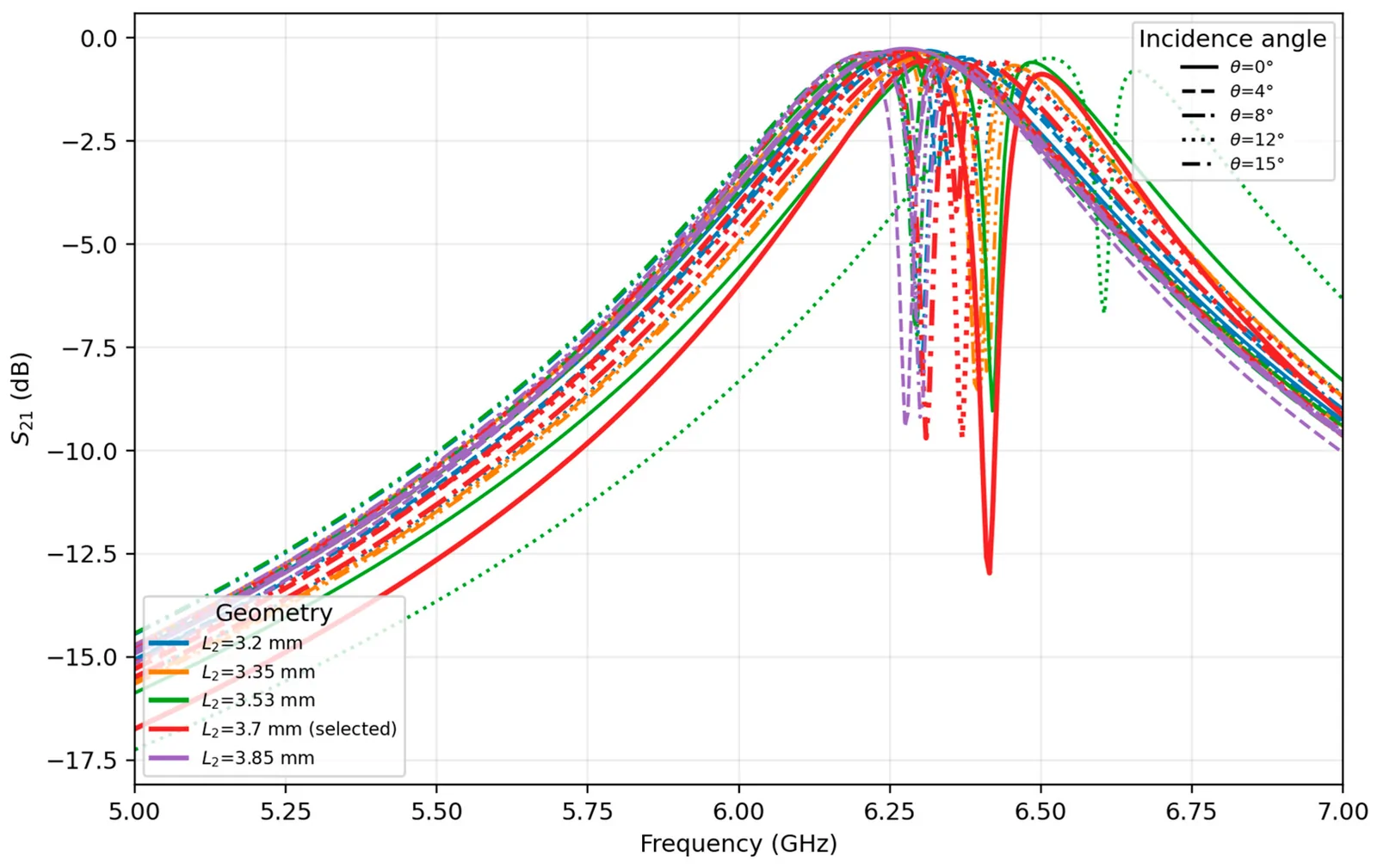
Influence of *L*_2_ on *S*_21_(*f*) after fixing *L*_1_ = 7.00 mm for *θ* = 0°, 4°, 8°, 12°, and 15°. The selected value is *L*_2_ = 3.70 mm. Colors denote different values of L_2_, while line styles denote the incidence angles θ = 0°, 4°, 8°, 12°, and 15°. All curves were reconstructed from the original exported HFSS data.

**Figure 3:**
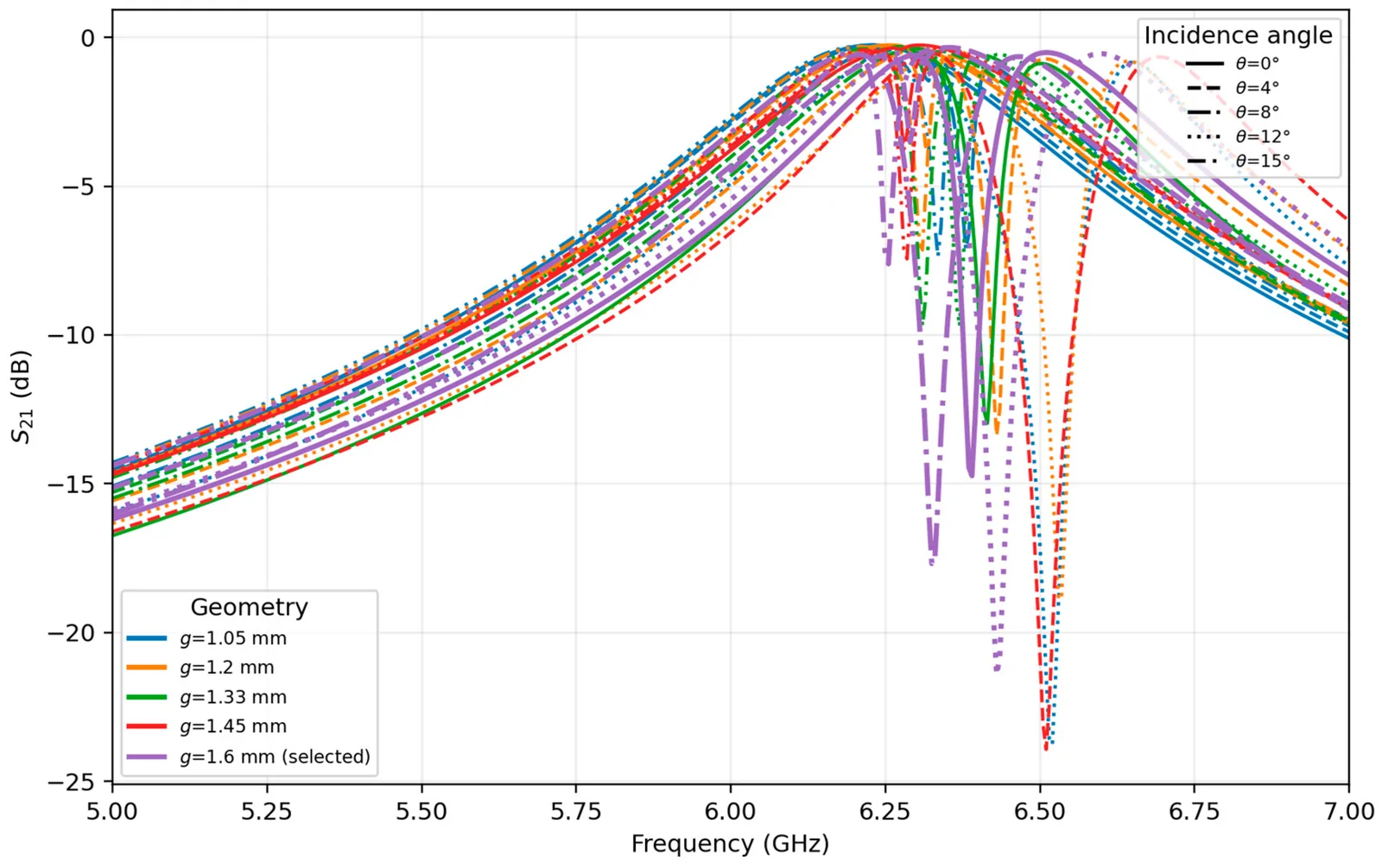
Influence of the central gap (*g*) on *S*_21_(*f*) after fixing *L*_1_ and *L*_2_ for *θ* = 0°, 4°, 8°, 12°, and 15°. The selected value is *g* = 1.60 mm. Colors denote different values of the central gap *g*, while line styles denote the incidence angles *θ* = 0°, 4°, 8°, 12°, and 15°. All curves were reconstructed from the original exported HFSS data.

**Figure 4:**
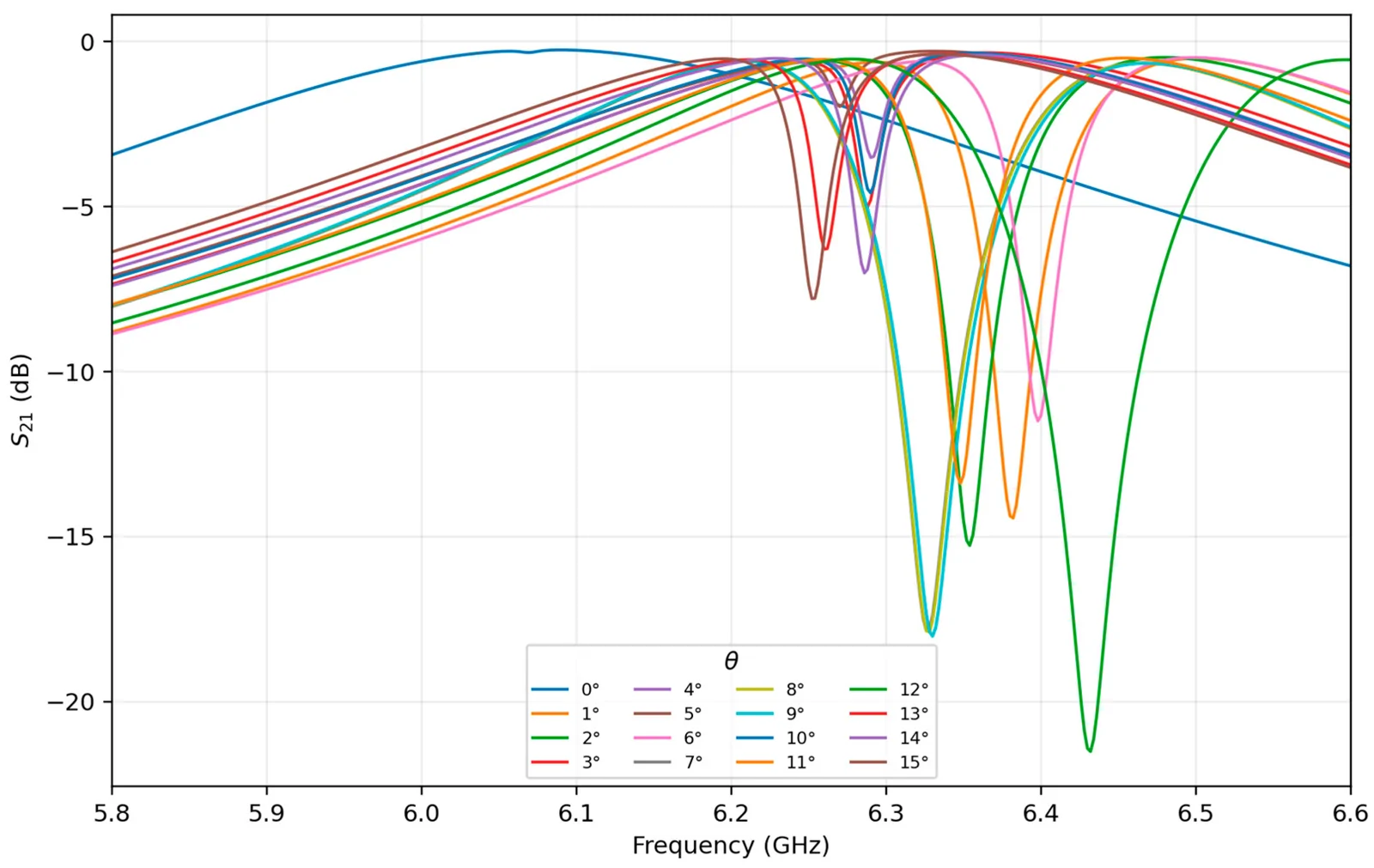
Frequency characteristics (*S*_21_(*f*)) of the selected metasurface for *θ* = 0–15° with a step of 1°. All curves are reconstructed from the original exported final HFSS angular sweep.

**Figure 5:**
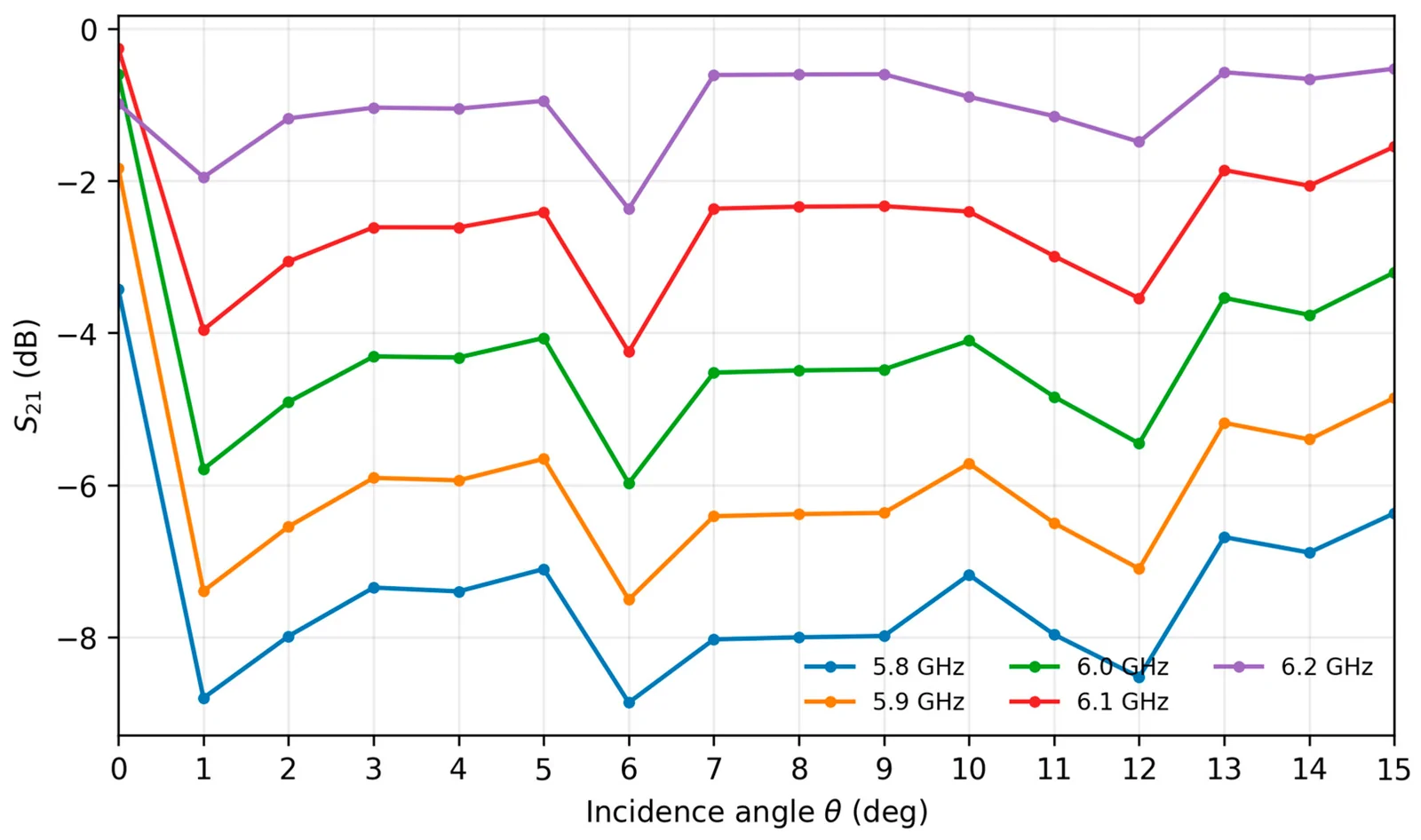
Angular dependences (*S*_21_(*θ*)) at 5.8, 5.9, 6.0, 6.1, and 6.2 GHz. Each colored curve corresponds to one fixed frequency. The curves were reconstructed from the exported HFSS data.

**Figure 6:**
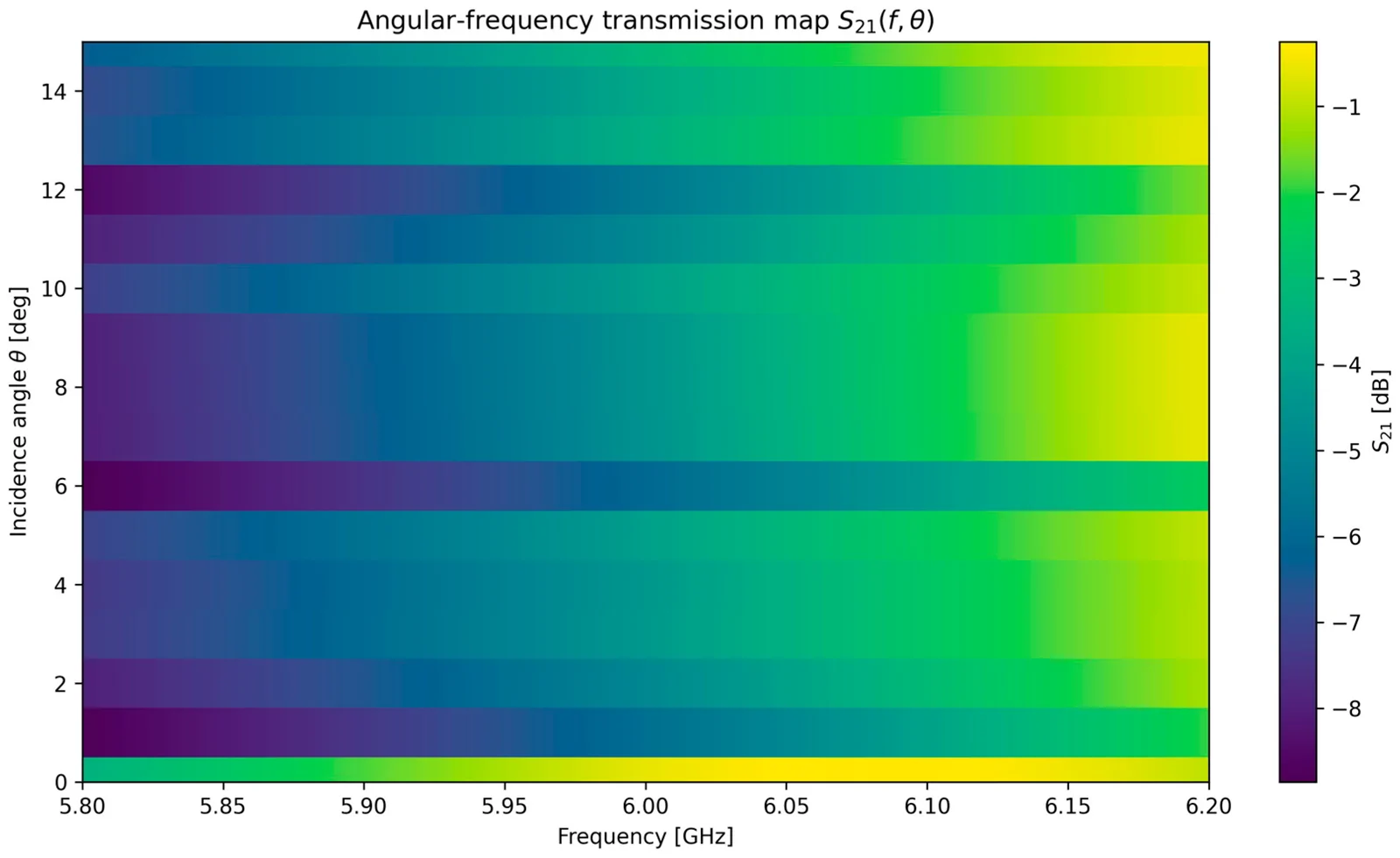
Two-dimensional representation of the *S*_21_(*f*, *θ*) characteristic calculated in HFSS in the operating band of 5.8–6.2 GHz.

**Figure 7:**
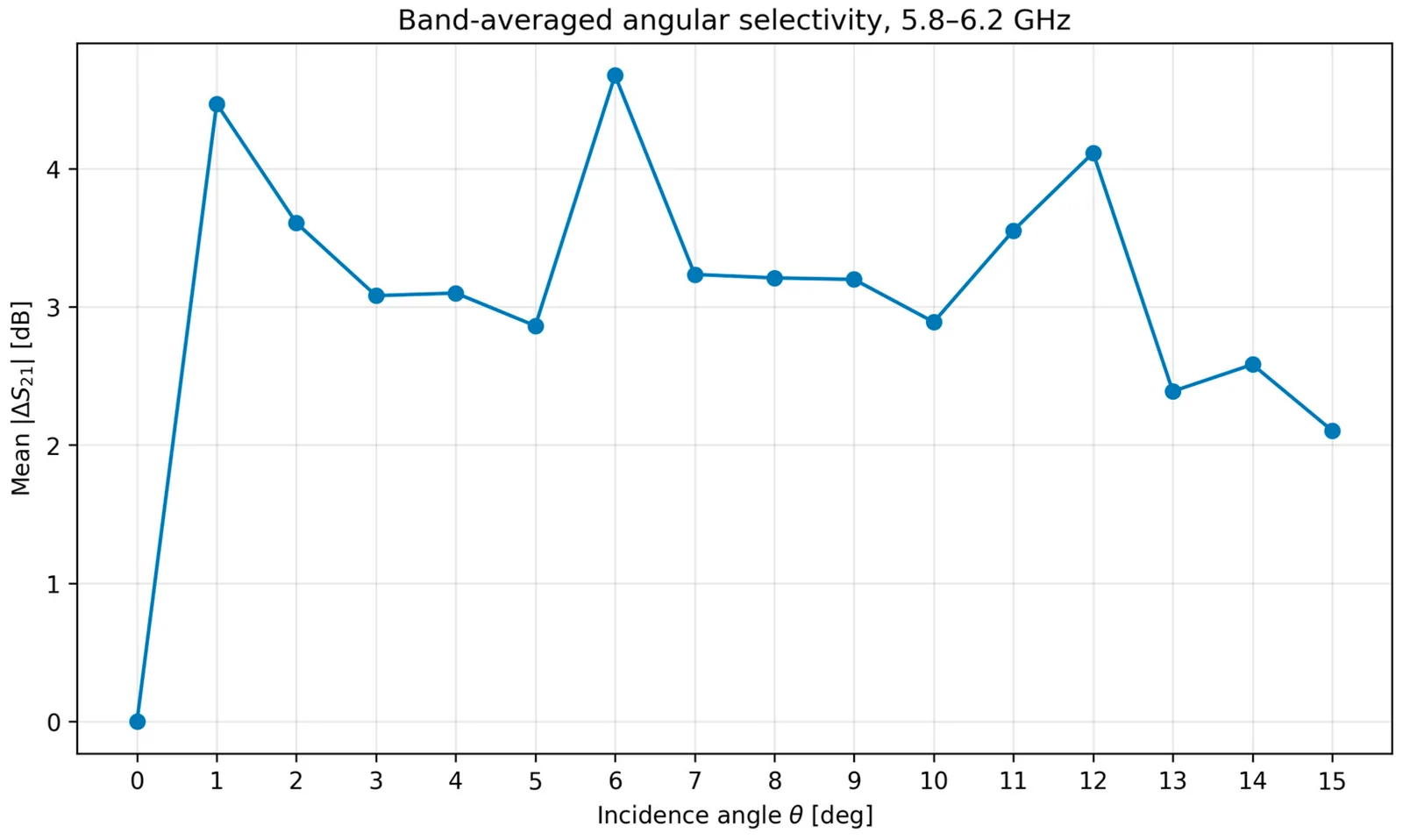
Average absolute change in S_21_ over the 5.8–6.2 GHz band relative to the normal incidence.

**Figure 8:**
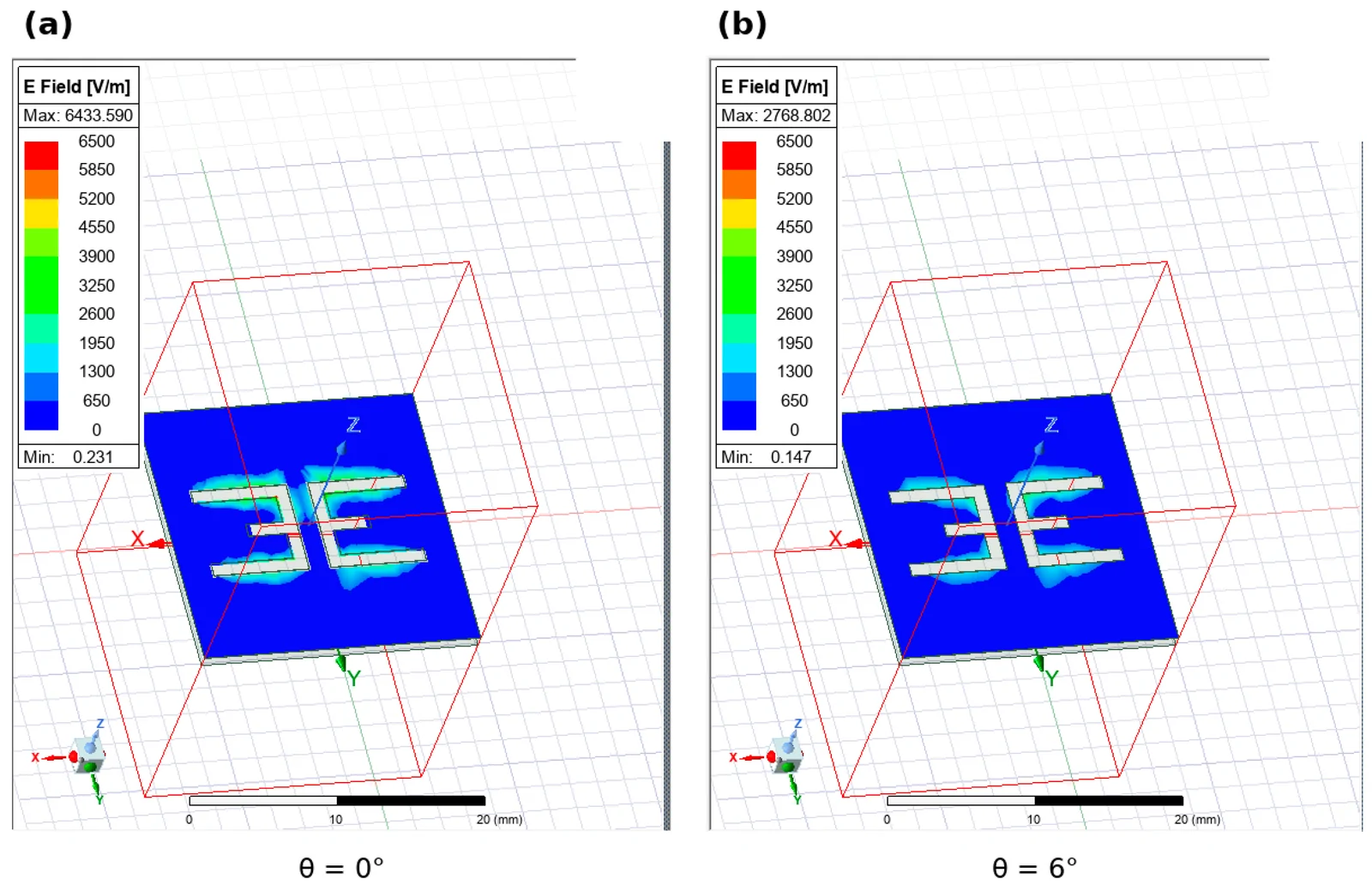
Simulated electric-field magnitude |*E*| in the substrate at 6.0 GHz: (**a**) *θ* = 0°; (**b**) *θ* = 6°. The same 0–6500 V/m scale is used for both panels.

**Table 1:** Geometric parameters of the optimized unit cell.

Parameter	Value
Cell period (*a*)	20.32 mm
Outer horizontal arm (*L*_1_)	7.00 mm
Inner horizontal arm (*L*_2_)	3.70 mm
Strip width (*w*)	0.97 mm
Center gap (*g*)	1.60 mm
Substrate thickness (*h*)	0.88 mm
Copper thickness (*t*Cu)	0.035 mm

**Table 2:** Comparison of this work with modern angle-selective and frequency-selective metasurfaces.

Reference	Frequency Domain	Angular Characteristic	Polarization	Verification	Purpose
Yan et al. [9]	7.2 GHz	−3 dB angular BW ≈ 8°	TE	Simulation + Experiment	Low-angle-pass filtering
Du et al. [10]	10 GHz	Pass 0–10°; stop 31–85°	Dual-polarized	Simulation + Experiment	Spatial filtering
Huang et al. [11]	15.56% of the relative bandwidth	High transmission ≈ up to 5°; suppression > 30°	Polarization-free	Simulation + Experiment	Broadband angular selection
Li et al. [12]	17.6–21.7 GHz (TE); 17.6–23 GHz (TM)	Ultra-narrow angular selectivity	Dual-polarized	Simulation + Experiment	Broadband ASS
Idrees et al. [13]	10 GHz	Angularly stable stopband	Polarization-insensitive	Simulation + Experiment	EMI shielding; a contrasting example of angle-stable FSS
Present work	5.8–6.2 GHz	Max. local contrast ≈ 5.67 dB; *θ* = 0–15°	Requires separate verification	Ansys HFSS	Physical selection and verification of trusted direction

## Data Availability

The original contributions presented in the study are included in the article; further inquiries can be directed to the corresponding authors.
